# Supplementary material for: Biosensors Based on Ion-Sensitive Field-Effect Transistors for HLA and MICA Antibody Detection in Kidney Transplantation
Source: Molecules. 2022 Oct 8;27(19):6697. doi: 10.3390/molecules27196697 (PMC9572778; doi:10.3390/molecules27196697)
Supplement: Supplementary file 1 [file molecules-27-06697-s001.zip › molecules-1952907-supplementary.pdf]

molecules

MDPI

Supplementary materials for the manuscript

ID: molecules-1375863

Title: Fabrication of Ion Sensitive Field Effect Transistor-based Biosensors for HLA and MICA Antibodies Detection in Kidney Transplantation

1. Limit of detection (LoD) and limit of quantitation

Table S1 The gate potential changes of the blank solution obtained from 10 individual ISFETs for each antibody

No.	Anti-HLA (0 μg/mL)		Anti-MICA (0 μg/mL)	
	ISFET ID	ΔV_{gs} of blank (mV)	ISFET ID	ΔV_{gs} of blank (mV)
1	D03_296	2.50	D03_462	5.10
2	D03_339	2.90	D03_465	4.40
3	D03_350	1.90	D03_484	7.06
4	D03_1567	1.80	D03_1490	4.32
5	D03_1568	3.50	D03_1258	6.01
6	D03_1478	2.60	D03_1259	3.00
7	D03_1479	1.40	D03_745	2.80
8	D03_1481	1.60	D03_746	5.04
9	D03_1483	2.10	D03_782	3.48
10	D03_1484	2.70	D03_808	1.10
	**Mean**	**2.30**	**Mean**	**4.23**
	**SD**	**0.65**	**SD**	**1.72**

1.1 Calculation of LoD

The LoD was calculated using the formula (1), LoD = 3σ/S;

$$LoD_{(Anti\text{-}HLA)} = 3(0.65)/0.9862 = 1.98\ \mu g/mL$$

$$LoD_{(Anti\text{-}MICA)} = 3(1.78)/0.9986 = 5.17\ \mu g/mL$$

1.2 Calculation of LoQ

The LoD was calculated using the formula (2), LoD = 10σ/S;

$$LoQ_{(Anti\text{-}HLA)} = 10(0.65)/0.9862 = 6.59\ \mu g/mL$$

$$LoD_{(Anti\text{-}MICA)} = 10(1.78)/0.9986 = 17.22\ \mu g/mL$$

2. Cut-off determination

Table S2 The gate potential changes of the isotype antibody (negative control) obtained from seven different concentrations of antibodies.

Isotype of anti-HLA		Isotype of anti-MICA	
Antibody Conc. (μg/mL)	$\Delta V_{gs\ (antibody)}$ of Isotype (mV)	Antibody Conc. (μg/mL)	$\Delta V_{gs\ (antibody)}$ of Isotype (mV)
0	2.43	0	5.52
5	2.63	5	5.43
10	2.70	10	6.33
20	3.33	20	6.65
40	3.49	40	7.19
80	3.40	80	7.47
160	3.93	160	7.43
**Mean**	**3.13**	**Mean**	**6.57**
**SD**	**0.55**	**SD**	**0.85**
**Cut-off**	**4.78**	**Cut-off**	**9.12**

Calculation of cut-off value

The cut-off value was calculated using the formula (3), Cut-off = $\alpha\ .\ \bar{X} + f\ .\ SD$;

$$\text{Cut-off}_{(\text{Anti-HLA})} = (1 \times 3.13) + (3 \times 0.55) = 4.78 \text{ mV}$$

$$\text{Cut-off}_{(\text{Anti-MICA})} = (1 \times 6.57) + (3 \times 0.85) = 9.12 \text{ mV}$$

3. Analytical precision

Table S3 The reproducibility of the ISFET-based immunosensor for anti-HLA and anti-MICA detection.

No.	anti-HLA (20 μg/mL)			anti-MICA (20 μg/mL)		
	ISFET ID	$\Delta V_{gs\ (antibody)}$	Antibody concentration from the inter-assay (μg/mL)	ISFET ID	$\Delta V_{gs\ (antibody)}$	Antibody concentration from the inter-assay (μg/mL)
1	D03_479	9.50	19.30	D03_1406	10.10	19.28
2	D03_480	10.60	22.76	D03_1420	10.08	19.20
3	D03_502	10.80	23.39	D03_1558	9.90	18.41
4	D03_503	11.12	24.40	D03_1060	10.30	20.16
5	D03_504	10.20	21.50	D03_1061	10.50	21.03
6	D03_1211	10.50	22.45	D03_1062	10.20	19.72
7	D03_1219	9.90	20.56	D03_1575	9.60	17.10
8	D03_1231	8.70	16.78	D03_1576	10.53	21.16
9	D03_1233	9.30	18.67	D03_1582	10.90	22.77
10	D03_1234	9.70	19.93	D03_1584	11.01	23.25
	**Mean**	**10.03**	**20.97**	**Mean**	**10.31**	**20.21**
	**SD**	**0.71**	**2.24**	**SD**	**0.41**	**1.80**
	**%CV**	**7.09**	**10.69**	**%CV**	**4.00**	**8.92**

4. Sensitivity and specificity in the experimental setting

Table S4 The sensitivity and specificity in the experimental setting for anti-HLA

	Anti-HLA			Isotype control	
**No.**	**ISFET ID**	**ΔV_{gs} after Ab binding (mV)**	**No.**	**ISFET ID**	**ΔV_{gs} after Ab binding (mV)**
1	D03_343	13	1	D03_550	3.2
2	D03_351	8	2	D03_553	2.6
3	D03_1614	8.5	3	D03_546	2.9
4	D03_244	2.9	4	D03_127	1.7
5	D03_248	3.8	5	D03_245	8.2
6	D03_258	4.4	6	D03_249	7.3
7	D03_139	10.41	7	D03_255	6.2
8	D03_147	9.81	8	D03_481	2
9	D03_117	1.2	9	D03_482	2
10	D03_477	8.92	10	D03_520	1.3
11	D03_479	9.5	11	D03_523	4.2
12	D03_480	10.6	12	D03_530	4.4
13	D03_502	10.8	13	D03_531	3.1
14	D03_503	11.12	14	D03_532	2.5
15	D03_504	10.2	15	D03_535	2.8
16	D03_1211	10.5	16	D03_584	2.7
17	D03_1219	9.9	17	D03_550	2.5
18	D03_1231	8.7	18	D03_1239	3.6
19	D03_1233	9.3	19	D03_1307	2.7
20	D03_1234	9.7	20	D03_1440	3.5
			21	D03_1441	3.4
			22	D03_1439	3.1
				**Sensitivity**	80.00%
				**Specificity**	86.36%

The cut-off value used to distinguish between positive and negative is 4.78 mV.
Immobilized-protein concentration is 10 μg/mL
The concentration of anti-HLA and isotype antibody control used in the experiment is 20 μg/mL.
The gray label showed the $\Delta V_{gs\ (Antibody)}$ that were less than or equal to the cut-off value (false negative).
The blue label showed the $\Delta V_{gs\ (Antibody)}$ that were greater than the cut-off value (false positive).

Table S5 The sensitivity and specificity in the experimental setting for anti-MICA

	Anti-MICA			Isotype control	
No.	ISFET ID	ΔV_{gs} after Ab binding (mV)	No.	ISFET ID	ΔV_{gs} after Ab binding (mV)
1	D03_1582	12.9	1	D03_914	8.26
2	D03_1584	11.2	2	D03_915	10.55
3	D03_804	9.5	3	D03_916	5.61
4	D03_837	12.3	4	D03_944	4.9
5	D03_876	11.4	5	D03_952	7.84
6	D03_893	12.5	6	D03_958	5.89
7	D03_901	15.32	7	D03_960	5.12
8	D03_905	11.6	8	D03_838	5.24
9	D03_910	18.5	9	D03_841	7.27
10	D03_744	12	10	D03_843	6.67
11	D03_747	11.1	11	D03_845	5.19
12	D03_765	13.46	12	D03_882	6.54
13	D03_782	9.11	13	D03_884	3.22
14	D03_794	12.47	14	D03_930	11.23
15	D03_801	10.55	15	D03_860	5.7
16	D03_828	11.96	16	D03_874	7.1
17	D03_807	11.3	17	D03_878	4.9
18	D03_808	13.81	18	D03_885	5.5
19	D03_815	6.6	19	D03_886	5.9
20	D03_816	11.2	20	D03_890	9.9
21	D03_829	10.2	21	D03_900	8.1
22	D03_832	11.4	22	D03_846	2.2
23	D03_839	13.1	23	D03_848	5.5
24	D03_849	12.6	24	D03_868	5.3
25	D03_746	11.8	25	D03_875	7.7
26	D03_755	8.5	26	D03_877	3.6
27	D03_769	13.8	27	D03_895	8.2
28	D03_820	7.1			
29	D03_826	12.2			
30	D03_834	12.4			
				**Sensitivity**	86.67%
				**Specificity**	88.89%

The cut-off value used to distinguish between positive and negative is 9.12 mV.
Immobilized-protein concentration is 50 μg/mL
The concentration of anti-HLA and isotype antibody control used in the experiment is 20 μg/mL.
The gray label showed the $\Delta V_{gs\ (Antibody)}$ that were less than or equal to the cut-off value (false negative).
The blue label showed the $\Delta V_{gs\ (Antibody)}$ that were greater than the cut-off value (false positive).
